# Editorial: Innovations and strategies for comprehensive frailty management in older people

**DOI:** 10.3389/fmed.2026.1790696

**Published:** 2026-02-17

**Authors:** Jie Li, Chengchao Zhou, Finbarr C. Martin, Miao Li

**Affiliations:** 1 School of Nursing, Tongji Medical College, Huazhong University of Science and Technology, Wuhan, China; 2 Shandong University, Jinan, China; 3 King's College London, London, United Kingdom

**Keywords:** comprehensive care, frailty assessment, frailty management, frailty prevention, multidisciplinary care models

As the global population ages at an accelerating pace, frailty has emerged as a central public health issue affecting the health and quality of life of older adults. As a complex multisystem syndrome characterized by diminished physiological reserve, frailty significantly increases the risk of adverse outcomes such as falls, disability, hospitalization, and mortality among older people. To advance integrated, evidence-based solutions, we launched the Research Topic “*Innovations and Strategies for Comprehensive Frailty Management in Older People*” in Frontiers in Medicine's Geriatric Medicine section. This Research Topic assembles 19 cutting-edge studies spanning original research, reviews, and practical interventions, conducting in-depth exploration around the four core dimensions of frailty management: mechanisms, assessment, interventions, and multidisciplinary models.

## Multidimensional mechanisms and risk factors of frailty

Frailty is not a monolithic condition of mere physical decline. A central theme emerging from this Research Topic is its profound complexity, arising from the intricate interplay of biological, psychological, and social determinants. Biologically, anemia emerges as a critical mediator linking frailty to adverse outcomes. Li, Zhao et al. conducted research based on data from the China Health and Retirement Longitudinal Study (CHARLS) and found that anemia mediates the association between frailty index (FI) and hip fractures, accounting for 18.95% of the total effect. Additionally, a single-center prospective study design proposed by Wang et al. examined how preoperative intrinsic capacity decline predicts post-operative frailty in elderly patients undergoing colorectal surgery, highlighting the role of preexisting physiological deficits in shaping frailty trajectories following surgical stress. Psychologically, depressive symptoms play a key mediating role in the association between frailty and multimorbidity. Geng et al. analyzed CHARLS data and found that depression served as a strong mediating factor, accounting for 35.20% of the effect between the multimorbidity patterns and physical frailty, rising to 69.84% among adults aged 60 and older. In specific subgroups with economic support or a high school education, depression fully mediated this relationship, suggesting that mental health interventions may be most impactful among socially advantaged groups. Su et al. explored the concept of self-perceived aging, demonstrating that positive self-perceived aging (SPA) reduces the risk of sarcopenia over 4 years, with instrumental activities of daily living (IADL) serving as a key behavioral mediating variable. Socially, Huang et al. revealed in a multi-center cross-sectional study that social frailty, as measured by the HALFT scale, significantly reduced the quality of life in patients with chronic heart failure. Family dysfunction and social networks, respectively, mediated 25.87% and 58.97% of this relationship. Beyond the aforementioned clinical insights, a narrative review by Li, Zhang et al. summarized the antioxidant, anti-inflammatory, and anti-osteoporotic effects of flavonoids, which exert anti-aging properties through mechanisms such as modulating the senescence-associated secretory phenotype (SASP).

## Innovations in frailty assessment and risk prediction

Accurate, standardized assessment is the cornerstone of effective frailty management. This Research Topic has achieved significant progress in the innovation, validation, and clinical translation of frailty measurement tools. A notable contribution is the development and validation of the Functional Limitations and Frailty in Geriatric Syndromes Questionnaire (FLIGS-FQ-16) by Bernardini et al. in a single-center study involving 900 elective and acute medical patients. This questionnaire demonstrates excellent discriminatory power, offering a practical solution for rapid clinical screening and stratification. Its focus on modifiable risk factors makes it particularly suitable for individualized treatment planning. Mangini et al. developed an Italian cross-cultural adaptation and validation of the Italian Social Vulnerability Index (SVI-I), which has provided a well-validated tool for identifying social deficits among community-dwelling older adults, enabling personalized interventions targeting the accumulation of physical, psychological, and social vulnerabilities.

Frailty prediction models achieve new levels of complexity through machine learning integration. Guo et al. developed a cognitive frailty score for older adults with multimorbidity in a tertiary hospital-based study, demonstrating robust discriminatory ability (C-index = 0.818, training AUC = 0.827, validation AUC = 0.784) and identifying six initial variables: drinking, constipation, polypharmacy, chronic pain, nutrition, and depression. Similarly, Yan et al.'s predictive model for frailty risk in older adults with cardiovascular-metabolic comorbidities, constructed using CHARLS data and incorporating depression, social activity, history of falls, life satisfaction, ADL scores, cognitive function, age and chronic disease burden, achieved an AUC of 0.816 in both training and validation sets with excellent calibration (Hosmer-Lemeshow *p* = 0.073 and 0.245). For community-living populations, Qi et al. demonstrated that machine learning algorithms (random forest and XGBoost) outperformed traditional decision trees in identifying Body mass index (BMI), living arrangements, visit frequency, and smoking status as key determinants for older adults in eastern China. For hospitalized frail elderly, the dysphagia risk model established by Lin et al. identified age, coughing history, polypharmacy, malnutrition, oral health-related self-efficacy, and oral health assessment index as key factors, with an AUC of 0.875.

Non-invasive biomarkers show promise for frailty risk stratification. A single-center prospective cohort study by Gao et al. demonstrated that the Systemic Immune Inflammation Index (SII) and Systemic Inflammatory Response Index (SIRI) can predict frailty progression in elderly patients undergoing elective orthopedic surgery, with a combined AUC of 0.723. Chen et al. validated that the geriatric nutritional risk index (GNRI) effectively predicts post-operative delirium (POD) in 820 patients undergoing revision arthroplasty.

## Targeted interventions for frailty management

Targeted interventions informed by precise assessment are pivotal to improving outcomes for frail older adults, and this Research Topic validates personalized, evidence-based strategies across clinical and community settings that address their biological, behavioral, and functional needs. A network meta-analysis by Liu et al. of 35 randomized controlled trials (*N* = 2,905) demonstrated that mind-body training (Tai Chi, Baduanjin) significantly alleviated frailty (SMD = −0.71, 95% CI: −1.22 to −0.21) and improved quality of life (SMD = 1.02, 95% CI: 0.89–1.15), outperforming aerobic and mixed training regimens.

Nutritional optimization emerges as a critical intervention. A retrospective analysis of prospectively collected data by Chatmongkolchart et al. demonstrated that combining the Clinical Frailty Scale (CFS) and the Prognostic Nutritional Index (PNI) improves the prediction of post-operative complications in non-cardiac surgery patients (AUC = 0.694), outperforming the use of either tool alone. For revision arthroplasty patients, optimizing GNRI above 101.96 reduces POD risk, highlighting the value of preoperative nutritional support. The single-center prospective study design proposed by Wang, Tian et al. aims to clarify whether preoperative intrinsic capacity decline predicts post-operative frailty in colorectal surgery patients, with the goal of guiding targeted preoperative optimization.

Psychosocial and resource-based strategies prove equally crucial. A cross-sectional study by Wang, Zhu et al. involving 627 Chinese stroke survivors demonstrated that family care not only directly enhances self-management behaviors but also exerts indirect effects by improving utilization of chronic disease resources. This underscores the importance of incorporating strengthened family support systems and actively promoting patient access to medical and social resources as integral components of comprehensive intervention programs.

Collectively, these studies emphasize that effective interventions must be tailored to frailty severity, comorbidities, and functional status.

## Multidisciplinary practice and system integration

Translating frailty research into clinical practice requires systematic integration across settings, and disciplines. Prehospital trauma triage represents a critical gap in frailty care. Harthi et al.'s two narrative reviews emphasize that older adults face disproportionately high rates of under-triage or over-triage due to age-related physiological changes, frailty, and polypharmacy, compounded by triage tools designed for younger populations. They proposed that incorporating frailty assessments, refining age-specific triage criteria, and enhancing paramedic education can improve the precision of prehospital trauma triage for older adults.

Multidisciplinary collaboration is emphasized across studies. Perioperative care requires coordination between surgeons, anesthesiologists, and geriatricians to optimize frailty and nutrition. Community care integrates primary care providers, social workers, and physical therapists to deliver exercise, social support, and nutritional interventions. Prehospital care relies on paramedics trained in geriatric trauma assessment.

This holistic approach ensures that biological, psychological, and social needs of frail older adults are addressed across care settings.

## Future directions and research priorities

Although this Research Topic has advanced the field of frailty management, several key gaps remain, pointing the way for future research. First, newly developed assessment tools require further validation and implementation across diverse healthcare settings and cultures to ensure generalizability. Second, it is crucial to conduct long-term follow-up on the intervention measures (especially mind-body training and nutritional optimization), which helps to evaluate their sustained efficacy in delaying the progression of frailty. Third, digital health technologies such as wearable devices for real-time frailty monitoring can be further personalized to enhance assessment accessibility and intervention adherence, but attention must be paid to addressing literacy, and privacy concerns. Fourth, solving health inequalities is crucial because older adults in rural areas face barriers to accessing multidisciplinary frailty care. Finally, more research is needed on the interaction between frailty and multiple comorbidities to develop personalized interventions for individuals at high risk of frailty.

This Research Topic provides a comprehensive overview of the evolving science of frailty, deepening our understanding of its complex mechanisms, refining assessment and prediction tools, proposing innovative personalized intervention strategies, and clarifying pathways for multidisciplinary collaboration. In the face of accelerating global aging, translating these insights into clinical practice and health policy is now imperative.

We extend our deepest gratitude to all authors, reviewers, and contributors. We remain committed to fostering interdisciplinary collaboration to address the evolving challenges of frailty in older adults and anticipate sustained breakthroughs in comprehensive frailty management in the coming years.

